# Mercury-Induced Externalization of Phosphatidylserine and Caspase 3 Activation in Human Liver Carcinoma (HepG_2_) Cells

**DOI:** 10.3390/ijerph2006030005

**Published:** 2006-03-31

**Authors:** Dwayne J. Sutton, Paul B. Tchounwou

**Affiliations:** 1 Molecular Toxicology Research laboratory, NIH-Center for Environmental Health, College of Science, Engineering and Technology, Jackson State University, 1400 Lynch Street, Box 18540 Jackson, Mississippi 39217, USA

**Keywords:** Mercury, Apoptosis, Flow cytometry, HepG_2_ cells, Caspase 3, Annexin V

## Abstract

Apoptosis arises from the active initiation and propagation of a series of highly orchestrated specific biochemical events leading to the demise of the cell. It is a normal physiological process, which occurs during embryonic development as well as in the maintenance of tissue homeostasis. Diverse groups of molecules are involved in the apoptosis pathway and it functions as a mechanism to eliminate unwanted or irreparably damaged cells. However, inappropriate induction of apoptosis by environmental agents has broad ranging pathologic implications and has been associated with several diseases including cancer. The toxicity of several heavy metals such as mercury has been attributed to their high affinity to sulfhydryl groups of proteins and enzymes, and their ability to disrupt cell cycle progression and/or apoptosis in various tissues. The aim of this study was to assess the potential for mercury to induce early and late-stage apoptosis in human liver carcinoma (HepG_2_) cells. The Annexin-V and Caspase 3 assays were performed by flow cytometric analysis to determine the extent of phosphatidylserine externalization and Caspase 3 activation in mercury-treated HepG_2_ cells. Cells were exposed to mercury for 10 and 48 hours respectively at doses of 0, 1, 2, and 3 μg/mL based on previous cytotoxicity results in our laboratory indicating an LD_50_ of 3.5 ± 0.6 μg/mL for mercury in HepG_2_ cells. The study data indicated a dose response relationship between mercury exposure and the degree of early and late-stage apoptosis in HepG_2_ cells. The percentages of cells undergoing early apoptosis were 0.03 ± 0.03%, 5.19 ± 0.04%, 6.36 ± 0.04%, and 8.84 ± 0.02% for 0, 1, 2, and 3 μg/mL of mercury respectively, indicating a gradual increase in apoptotic cells with increasing doses of mercury. The percentages of Caspase 3 positive cells undergoing late apoptosis were 3.58 ± 0.03%, 17.06 ± 0.05%, 23.32 ± 0.03%, and 34.51 ± 0.01% for 0, 1, 2, and 3 μg/mL of mercury respectively, also indicating a gradual increase in Caspase positive cells with increasing doses of mercury.

## Introduction

All forms of mercury are toxic and exert their effects in a number of organs, tissues, and cell systems [1]. Mercury is of significant concern as an environmental pollutant because it is stable, persistent, and cannot be degraded or destroyed [2]. Mercury and its compounds both organic and inorganic are released to the environment as a result of a variety of human activities. Therefore, it tends to accumulate in the soils and sediments [3]. Excessive levels of mercury in the marine environment can affect marine biota and pose risk to human consumers of seafood. Hence, mercury compounds found in the marine environment pose risk to human health through the consumption of contaminated seafood. Accordingly, it is desirable to minimize such exposure to levels that do not cause adverse effects [4].

Previous research has documented that mercury is cytotoxic [5]. Its biochemical effects at the cellular level include DNA damage, and inhibition of DNA and RNA synthesis [6]. Mercury also causes alteration in protein structure, alterations in calcium transport, along with inhibition of glucose transport and enzyme function [7].

The toxicokinetics (i.e., absorption, distribution, metabolism, and excretion) of mercury is highly dependent on the form of mercury to which a receptor has been exposed. The absorption of inorganic mercury through the gastrointestinal tract varies with the particular mercuric salt involved. The absorption decreases with decreasing solubility. Estimates of the percentage of inorganic mercury that is absorbed vary; as much as 20% may be absorbed. Inorganic mercury has a reduced capacity for penetrating the blood-brain or placental barriers. However, there is some evidence indicating that mercuric mercury in the body following oral exposures can be reduced to elemental mercury and excreted via exhaled air. Because of the relatively poor absorption of orally administered inorganic mercury, the majority of the ingested dose in humans is excreted through the feces [8].

There are many signs and symptoms of mercury toxicity. Toxicity can be associated with four categories of mercury; metallic or elemental mercury, which is relatively mild; inorganic mercury, such as mercury chlorides, which primarily affect the liver/kidneys; organo-mercurials, such as mercury salts in diuretics or fungicides, which convert to inorganic mercury; and short chain alkyl mercury compounds, of which methyl mercury is the most toxic [9]. Mercury exposure induces a significant number of adverse health effects including: cardiovascular disease, anemia, developmental abnormalities, neurobehavioral disorders, kidney and liver damage, and cancer in some cases. In several studies, the toxicity of mercury has been attributed to its high affinity to protein-containing sulfhydryl groups. Based on the chemical, biological, and environmental characteristics of the various forms of mercury, it has been established that inorganic mercury, is the form most likely to pose a hazard in drinking waters

One of the mechanisms by which mercury exerts its toxic effect is through impairment of cellular respiration by the inhibition of various mitochondrial enzymes, and the uncoupling of oxidative phosphorylation. Most toxicity of mercury results from a direct mechanism involving mercury’s inhibition of cellular enzymatic processes by binding with the hydroxyl radical (SH) in amino acids [10]. This appears to also play a major part in mercury’s ability to elicit allergic/immune reactive conditions [11]. Additional cellular level enzymatic effects of mercury binding with proteins include the blockage of sulfur oxidation processes [12], enzymatic processes involving vitamins B6 and B12 [13], effects on cytochrome-c energy processes [14], along with mercury’s adverse effect on the cellular mineral levels of calcium, magnesium, zinc, and lithium [15]. Mercury has also been found to cause additional neurological immune systems effects through immune and autoimmune reactions [16].

Previous studies have demonstrated that mercury is cytotoxic and induces apoptosis in various cells lines. Therefore, the goal of this study was to assess the potential mechanisms of mercury induced apoptosis in HepG_2_ cells.

## Materials and Methods

### Chemical and Growth Medium

Reference solution of mercury (10,000 μg/mL), CAS No. 7439-97-6 Lot No. B0095024 was purchased from EM Science (Gibbstown, New Jersey). Dulbecco’s Modified Eagle’s Minimal Essential Medium was purchased from Life Technologies (Grand Island, New York). Penicillin-Streptomycin, Lot No. 1085899 and fetal bovine serum (FBS) were purchased from BRL Technologies (Grand Island, New York)

### Cell Culture

Transformed human hepatoma cells (HepG_2_ ATCC No. 8065) cells were purchased from American Type Culture Collection, (Manassas, VA). In the laboratory, cells were stored in liquid nitrogen until use. They were next thawed by gentle agitation of their containers (vials) for 2 minutes in a water bath at 37°C. After thawing, the content of each vial was transferred to a 75 cm^2^ tissue culture flask, diluted with DMEM, supplemented with 10% fetal bovine serum (FBS), 1% streptomycin and penicillin, and incubated for 24 hours at 37°C in a 5% CO_2_ incubator to allow the cells to grow, and form a monolayer in the flask. Flasks were then treated with 1, 2, and 3ppm of mercury for 10 and 48 hours, and a control that received no treatment.

### Flow Cytometric Analysis of Phosphatidylserine Externalization

The percentage of apoptotic HepG_2_ cells were determined by analyzing phosphatidylserine exposure and membrane integrity by double staining with a FITC-conjugated Annexin V antibody and propidium iodide (PI) (1μg/ml) ((BD Biosciences, San Jose CA) using flow cytometric analysis (FACSCalibur and Cell Quest Pro Software, BD Biosciences, San Jose, CA). HepG_2_ cells were exposed to mercury for a period of 10 hours. Cells (10^6^). were washed with PBS, re-suspended in 50 μl of 1X binding buffer and then incubated 10 minutes at room temperature in the dark The cells were then washed and re-suspended in binding buffer and flow cytometric analysis was performed within 30 minutes of staining using FL1 versus FL2 dot plot analysis acquiring 10,000 events. The instrument settings were optimized using a negative control of untreated HepG_2_ cells and a positive control of camptothecin treated HepG_2_ cells. Results were analyzed and statistical analysis done using the Cell Quest Pro software BD Biosciences.

### Flow Cytometric Analysis of Caspase 3 Activation

The percentage of Caspase 3 active HepG_2_ cells exposed to mercury was determined by a single staining with a Caspase 3 Antibody (BD Biosciences, San Jose, CA) and using flow cytometric analysis (FACSCalibur and Cell Quest Pro Software, BD Biosciences, San Jose, CA). Cells (10^6^) were washed twice with PBS, re-suspended in 50 μl of 1X binding buffer and incubated on ice for 20 minutes. Cells were then washed twice if Perm/Wash buffer (0.5 ml). Cells were then re-suspended in Perm/Wash buffer and antibody for thirty minutes at room temperature. The cells were then washed and re-suspended in binding buffer and flow cytometric analysis were performed within 30 minutes of staining using FL1 versus FL2 dot plot analysis acquiring 10,000 events. The instrument settings were optimized using a negative control of untreated HepG_2_ cells and a positive control of camptothecin treated HepG_2_ cells. Results were analyzed and statistical analysis done using the Cell Quest Pro software BD Biosciences.

## Results and Discussion

**Figure 1:**
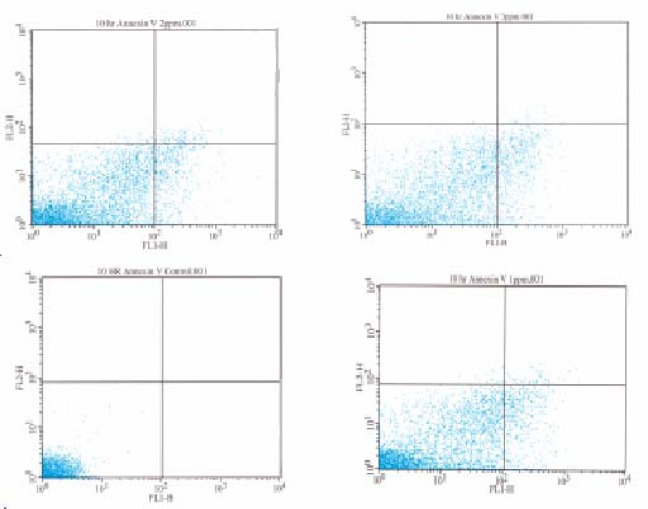
Flow cytometric analysis of Annexin-V activity in HepG_2_ cells exposed to mercury

Figure 1 shows the dot plot analysis in which the Annexin positive cells are shown in the lower right quadrant. As the mercury dose increases, the number of Annexin positive cells increases.

**Figure 2:**
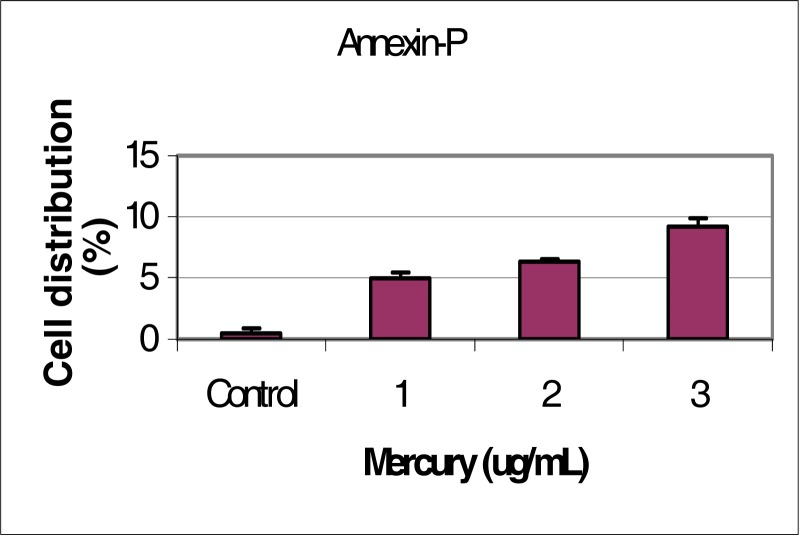
Proportion of Annexin V positive HepG_2_ cells in response to mercury exposure.

Figure 2 shows the percentage of Annexin-V Positive cells in response to mercury concentrations.

**Figure 3:**
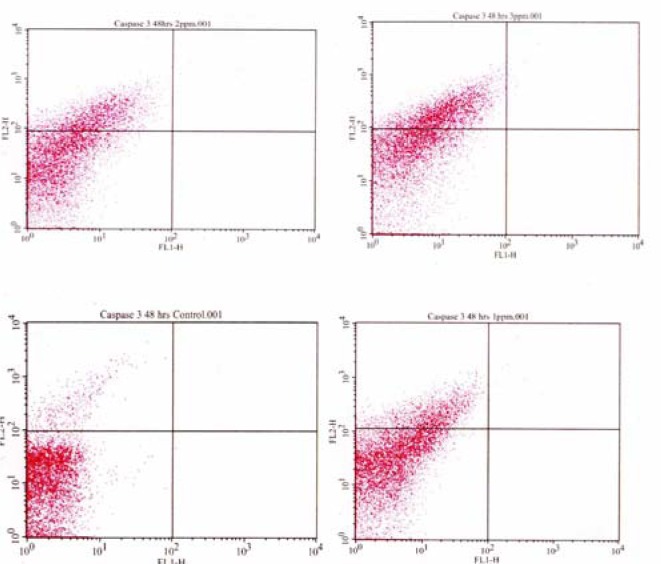
Flow cytometric analysis of Caspase 3 activity in HepG_2_ cells exposed to mercury.

Figure 3 shows the proportion of Caspase 3 positive HepG_2_ cell in response to mercury exposure. The percentage of cells undergoing late apoptosis were 3.58 ± 0.03%, 17.06 ± 0.05%, 23.32 ± 0.03%, and 34.51 ± 0.01% in 0, 1, 2, and 3μg/mL of mercury respectively; indicating a gradual increase in Caspase 3 activation with increasing doses of mercury. These data indicate that mercury induces late stage apoptosis in human liver carcinoma cells, in a dose dependent manner. These findings are in support of published literature reported mercury-induced apoptosis in other cell types [17].

**Figure 4:**
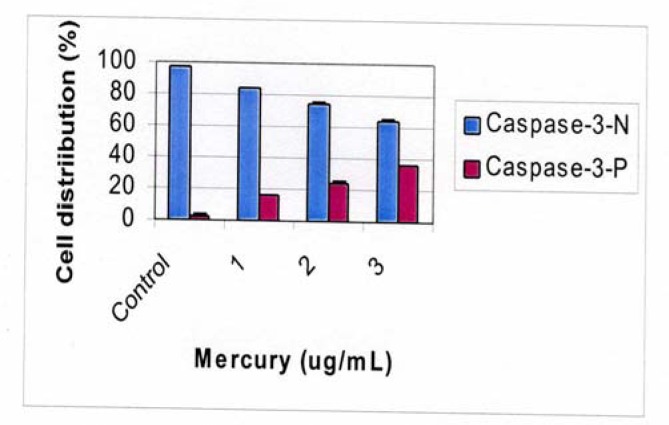
Proportion of Caspase 3 positive HepG_2_ cells in response to mercury exposure.

Figure 4 shows a dose dependent response of Caspase 3 positive cells in response to mercury exposure. The percentage of Caspase 3 positive cells increases as the concentration of the mercury increases in a dose dependent manner.

Cellular responses to environmental toxicants such as mercury are very much dependent on the cell line and degree of differentiation. Hence, cell death may occur in dividing cells because of unbalanced mitogenic stimulation. But the same conditions may not cause cytotoxicity in differentiated cells [18]. Inorganic mercury has been shown to change the fate of murine central nervous system stem cells, alter neuronal differentiation, affect neurotransmitter production, alter the conductance of L-type calcium channels, potentiate calcium signals elicited by depolarization or agonist stimulation, enhance NGF-induced differentiation in PC-12 cells, and modify calcium signals, thereby activating apoptosis in cell lines [19, 20, 21, 22]. Mercury has also shown the ability to cause changes on the cellular level in platelets and erythrocytes. These cells have been used as surrogate markers for mercury damage in neurological tissues, and it was found that the addition of methylmercury to whole blood caused a dramatic dissolution of microtubule platelets and red blood cells. This effect was more pronounced in erythrocytes than platelets which is consistent with the sequestration of mercury in erythrocytes [23, 24]. This effect on microtubules has also been shown to result in the disruption of the cell cycle and to lead to apoptosis in blood and neuronal cells, as well as other cell lines [25]. Our findings indicate that early stage apoptosis (the externalization of phosphatidylserine), and late stage apoptosis (Caspase 3 activation) is induced in human liver carcinoma (HepG_2_) cells exposed to mercury in a dose dependent manner.
